# Supplementary material for: YAMDA: thousandfold speedup of EM-based motif discovery using deep learning libraries and GPU
Source: Bioinformatics. 2018 May 22;34(20):3578–80. doi: 10.1093/bioinformatics/bty396 (PMC6184538; doi:10.1093/bioinformatics/bty396)
Supplement: Supplementary Data [file bty396_supplementary.docx]

Figure S1. Strip plots of TOMTOM motif comparisons of each of the YAMDA-derived motifs (x-axis labels) queried against JASPAR CORE 2018 vertebrate non-redundant motifs (719) and Homer ChIP-seq motifs (387). Each point corresponds to a -log_10_ TOMTOM p-value describing the similarity between the query motif and one of the target database motifs. Several points are highlighted with sequence logos from the database motifs. The JASPAR IRF4 motif is derived from SELEX data instead of ChIP-seq data and is a much poorer match for the YAMDA-derived IRF4 motif than the JASPAR IRF1 motif is. Homer’s IRF4 motif is derived from ChIP-seq data, but only captures one of the half-sites.
